# Oxidation sensitizes TRPV2 to chemical and heat stimuli, but not mechanical stimulation

**DOI:** 10.1016/j.bbrep.2021.101173

**Published:** 2021-11-18

**Authors:** Mai Oda, Yuichiro Fujiwara, Yasuki Ishizaki, Koji Shibasaki

**Affiliations:** a Department of Molecular and Cellular Neurobiology, Gunma University Graduate School of Medicine, Maebashi, 371-8511, Japan; b Molecular Physiology & Biophysics, Faculty of Medicine, Kagawa University, Kagawa, 761-0793, Japan; c Laboratory of Neurochemistry, Graduate School of Human Health Science, University of Nagasaki, 1-1-1 Manabino, Nagasaki, 851-2195, Japan

**Keywords:** 2-APB, 2-aminoethoxydiphenyl borate, ChT, chloramine T, LPC, lysophosphatidylcholine, ROS, reactive oxygen species, TRP, transient receptor potential, TRPV, transient receptor potential vanilloid, TRP channel, TRPV2, Oxidation, Heat, Mechanical stimulus, Two-electrode voltage clamp method

## Abstract

The transient receptor potential vanilloid 2 (TRPV2) ion channel is activated by a chemical ligand (2-aminoethoxydiphenyl borate; 2-APB), noxious heat and mechanical stimulation. In a heterologous mammalian cell expression system, the oxidant chloramine T (ChT) sensitizes TRPV2 activation in response to 2-APB and heat by oxidation of methionine residues at positions 528 and 607 in rat TRPV2. Here, we used a *Xenopus* oocyte expression system to determine whether ChT-mediated oxidation can also sensitize TRPV2 to mechanical stimulation. In this system, we confirmed that ChT sensitized TRPV2 activation in response to 2-APB and heat, but we detected no sensitization to mechanical stimulation. This result suggests that the activation mechanism of TRPV2 by a chemical ligand and heat differs from that for mechanical stimulation. Further, we demonstrated that two-electrode voltage clamp recording in the *Xenopus* oocyte expression system is an excellent format for high throughput analysis of oxidization of redox-sensitive TRP channels.

## 1 Background

The ability to sense sensory information such as chemical ligands, temperature and mechanical stress is crucial for survival of organisms. Such sensory information is detected by specialized primary afferent neurons that innervate the skin and produce signals that are transmitted through central projections to the spinal cord and the brain [1]. During sensory perception, cation channels allow Na ^+^ influx to induce depolarizing currents in somatosensory neurons. Extracellular Ca^2+^ influx is mediated by plasma membrane Ca^2+^-permeable cation channels, which act as sensors by converting cellular stimuli into chemical signals such as depolarization of membrane potential and changes in intracellular Ca^2+^ concentration ([Ca^2+^]i) [2]. Transient receptor potential (TRP) channels typically have high Ca^2+^ permeability and are a major ion channel family that detect noxious stimulation.

TRP channels are a large group of ion channels that are divided into six subfamilies (TRPC, TRPV, TRPM, TRPA, TRPML, and TRPP); in humans 27 different TRP channels have been identified [2,3]. TRP channels are expressed on various cells including neuronal and immune cells, and play an important role in sensing various stimuli including chemical ligands, temperature, mechanical stress and osmotic pressure [[4], [5], [6], [7]]. Among the TRP channels, the TRP vanilloid (TRPV) subfamily has six members (TRPV1-TRPV6) in mammals, including TRPV1, which is cloned as a vanilloid receptor that is activated by capsaicin, the pungent component of chili peppers [[8], [9], [10]].

TRPV2 is a non-selective cation channel identified as an orthologous gene of TRPV1 and functions as a noxious heat sensor with an activation threshold of >52 °C when expressed in a heterologous cell system [[9], [10], [11]]. However, the thermal responses of TRPV2 knockout mice are similar to those of wild-type mice in behavioral experiments, and thus the physiological role of TRPV2 in temperature sensing is unclear [12,13]. TRPV2 is also activated by chemical ligands such as cannabidiol, 2-aminoethoxydiphenyl borate (2-APB) and lysophosphatidylcholine (LPC) [[14], [15], [16], [17]]. We previously reported that TRPV2 expression occurs in dorsal root ganglion (DRG) neurons at embryonic day 10 (E10) and developing neurons promote axonal outgrowth by sensing membrane stretch via TRPV2 [18]. Furthermore, we found that mechanical stimulation perturbs the cytoskeletal architecture (actin filaments) and subsequent perturbations lead to additional TRPV2 activation and simultaneous TRPV2 accumulation [19]. We also demonstrated the molecular mechanism by which TRPV2 activation enhances growth cone motility and actin reorganization to promote axonal outgrowth [19]. These results strongly support a role for TRPV2 as a mechanosensitive and heat-sensitive ion channel [10].

Animals living in an aerobic environment are constantly faced with oxidative stress. The redox state in the body depends on a balance between the levels of intracellular antioxidants and redox reactive species such as reactive oxygen species (ROS). In general, the disruption of cellular redox homeostasis due to excessive production of redox reagents causes damage to membrane lipids, proteins, and DNA [20]. However, redox reactive species have recently been reported to play a role as signaling molecules that regulate biological and physiological processes [21]. ROS can sensitize the responsiveness of several TRP channels to chemical ligand and temperature. Some TRP channels, including TRPV1, TRPM2, TRPC5 and TRPA1 have sensitivity to redox agents such as H_2_O_2_, and have been shown to induce physiological phenomena such as inflammatory responses and vasodilation [[22], [23], [24], [25], [26], [27], [28]]. For example, TRPM2, which has an activation threshold of >48 °C, is activated by H_2_O_2_ produced by macrophage immune responses, and oxidation of methionine (M) residues in TRPM2 lowers its activation threshold below that of normal body temperature [25]. Oxidation of residues M528 and M607 in rat TRPV2 by the oxidant chloramine-T (ChT) was reported to sensitize this channel to 2-APB and heat [29]. Redox-sensitive TRP channels, including TRPV2 and TRPC5, are also activated by mechanical force [18,19,30], but whether oxidation enhances the sensitivity of mechanosensitive TRP channels is unclear. Moreover, most studies that examined the oxidation-dependent activation mechanism of TRP channels have mainly been conducted using primary cell culture systems and mammalian cell expression systems with cells lines like HEK293. The effect of oxidation on the chemical and heat sensitivity of TRP channels heterologously expressed in *Xenopus* oocytes, which have a different plasma membrane composition than cultured cells, has not been characterized.

In this study, we investigated whether oxidation by ChT increases TRPV2 sensitivity to the chemical ligand 2-APB and heat stimulation in *Xenopus* oocytes. We also examined whether ChT alters TRPV2 sensitivity to mechanical stimulation using a newly-designed mechanical stimulation system that allows examination of activation mechanisms similar to those seen for chemical ligands and heat.

## 2 Materials and methods

### 2.1 Experimental animals

All animal experiments were performed according to the guidelines of the Gunma University Animal Care, the University of Nagasaki Animal Care and Experimentation Committees. *X. laevis* were purchased from Hamamatsu Seibutsu Kyozai (Hamamatsu, Japan).

### 2.2 In vitro transcription

Full-length mouse TRPV2 was excised from a pcDNA3 plasmid by *Eco*RI and *Xba*I digestion. The resulting TRPV2 fragment was subcloned into a pGEMHE vector [31]. *mTRPV2* complementary RNA (cRNA) for oocyte injection was transcribed with a mMESSAGE mMACHINE T7 transcription kit (Ambion) according to the manufacturer's instructions.

### 2.3 Two-electrode voltage clamp method

After injection with 50 nl *TRPV2* cRNA (500 ng/μl), oocytes were incubated at 17 °C for 4–6 days. Ionic currents were recorded using an OC-725C amplifier (Warner Instruments) and digitized using Digidata 1440 (Axon Instruments). The recording bath solution contained 96 mM NaCl, 2 mM KCl, 1 mM MgCl_2_, 1.8 mM CaCl_2_ and 5 mM HEPES at pH 7.4 (adjusted with NaOH). Oocytes were voltage-clamped at −60 mV, and current data was obtained every 1 s. Chloramine T (ChT; MP Biomedicals) was dissolved in the bath solution. For heat stimulation, bath solution heated with a temperature controller (TC-344B; Warner Instruments) was applied by perfusion. For poking assay, mechanical stimulation was applied using a tungsten needle (outer diameter, 40 μm) mounted on the manual manipulator (NARISHIGE NMN-21). The needle set to 40° from the horizontal plane into the moving parts of manipulator. The tip of the needle was positioned through the rotation of dials so that it just attached to the cell membrane. After that, we rotated the dials (Z-axis) of manipulator (2.5 rotation). The needle was then moved toward the oocyte as the 8 μm step. It takes for 30 s that the needle moved 8 μm.

### 2.4 Statistical analysis

Data values are expressed as means ± SEM. Significance of the observed changes was assessed using Student's unpaired *t*-test and Tukey-Kramer method. A difference of p < 0.01 was considered to be statistically significant.

## 3 Results

### 3.1 ChT enhances 2-APB-evoked currents

**Fig. 1:**
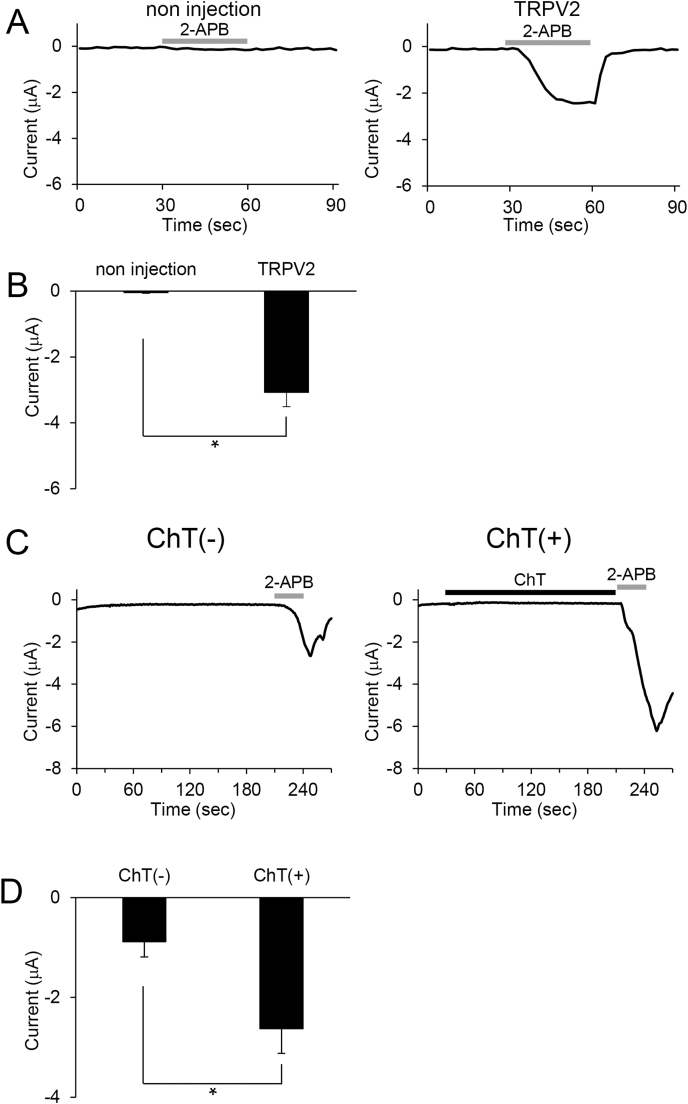
ChT potentiates 2-APB-evoked currents in TRPV2-expressing oocytes. (A) Representative traces of 2-APB evoked current in *Xenopus* oocytes without cRNA injection or expressing TRPV2. The gray bar indicates application of 3 mM 2-APB at 30 s. (B) Peak average currents after 2-APB application. Bars represent the mean ± SE (n = 4). *p < 0.05 (*t*-test). (C) Representative traces of 2-APB evoked current in *Xenopus* oocytes expressing TRPV2 without ChT (Left) or after treatment with 1 mM ChT for 3 min (Right, black bar). The gray bar indicates application of 3 mM 2-APB at 210 s. Measurements were collected at room temperature. (D) Average currents at 240 s (30 s after 2-APB application) without and with ChT treatment. Bars represent the mean ± SE (n = 5). *p < 0.05 (*t*-test).

We used a two-electrode voltage clamp method to confirm the functional expression of mouse TRPV2 (mTRPV2) in *Xenopus* oocytes (Fig. 1A). We cloned the mTRPV2 cDNA into the oocyte expression vector pGEMHE and injected *mTRPV2* cRNA to *Xenopus* oocytes. We then measured the inward current in mTRPV2-expressing oocytes after application of the TRPV2 agonist, 2-aminoethoxydiphenyl borate (2-APB) four days after injection to confirm that TRPV2 was functionally expressed in *Xenopus* oocytes. Although researchers usually use over 1 mM 2-APB for the TRPV2 activation [18,29], the mTRPV2-expressing oocytes did not show channel activation at 1 mM 2-APB (data not shown). This might be due to the penetrating rate of 2-APB depending on the specific plasma membrane structures of *Xenopus* oocytes. Since control oocytes without injection of cRNA did not respond to 3 mM 2-APB (Fig. 1A and B), this concentration was used for subsequent experiments. Functional expression of mTRPV2 in *Xenopus* oocytes was confirmed by the detection of inward currents following application of 3 mM 2-APB (Fig. 1A, right side. Fig. 1B). The 2-APB activated currents was abolished by the washout of the reagent (Fig. 1A, right side). These data indicate that *Xenopus* oocytes expressing mTRPV2 is useful for functional analysis of this channel.

We next examined the effect of ChT on 2-APB-evoked currents in *Xenopus* oocytes expressing mTRPV2. After treating the oocytes with 1 mM ChT for 3 min, 2-APB was applied. The mTRPV2-expressing oocytes were only minimally activated by application of 1 mM ChT alone, but upon application of 2-APB the mTRPV2-mediated inward current was dramatically enhanced (Fig. 1A, right side). Compared with non-treated oocytes, ChT treatment led to a 3-fold increase in the magnitude of 2-APB-induced TRPV2 currents (Fig. 1B). Thus, ChT appears to sensitize TRPV2 sensitivity to a chemical ligand, 2-APB, in the *Xenopus* oocyte expression system.

### 3.2 ChT sensitizes TRPV2 sensitivity to heat stimulation

**Fig. 2:**
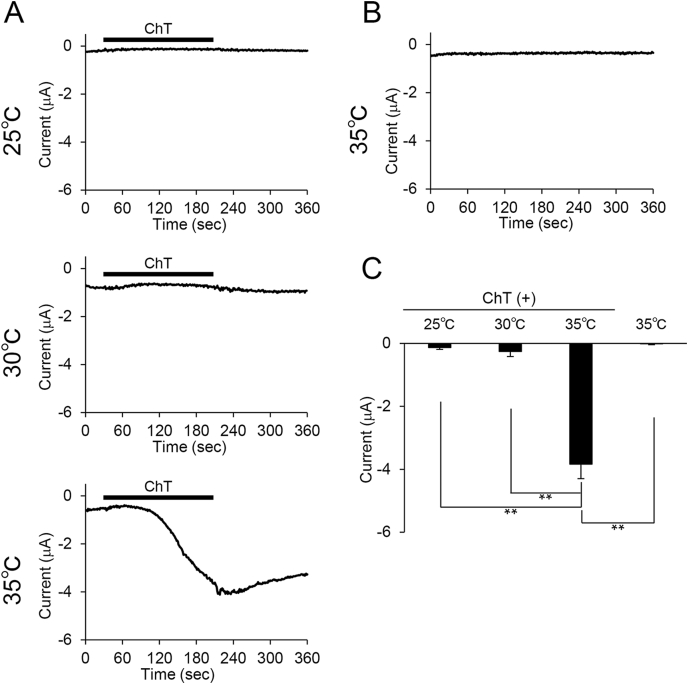
ChT affects heat sensitivity of TRPV2-expressing oocytes. (A) Representative traces of heat-evoked current in *Xenopus* oocytes expressing TRPV2 after treatment with 1 mM ChT for 3 min at 25 °C (Upper), 30 °C (Middle) or 35 °C (Bottom). Heat stimulation was applied from 0 s (B) Representative trace of heat-evoked currents in *Xenopus* oocytes expressing TRPV2 at 35 °C. Heat stimulation was applied from 0 s. (C) Peak average currents without ChT at 35 °C (far right) and after ChT treatment at 25 °C, 30 °C or 35 °C. Bars represent the mean ± SE (n = 5). **p < 0.01 (Tukey-Kramer method).

We observed significantly enhanced TRPV2 activity in response to 2-APB after ChT treatment, whereas application of 2-APB alone evoked only slight TRPV2 activation in our oocyte expression system (Fig. 1C and D). To further investigate the effect of ChT, we next examined whether ChT sensitizes heat-evoked currents in *Xenopus* oocytes expressing mTRPV2. We measured heat-evoked currents in oocytes treated for 3 min with 1 mM ChT at either 25 °C, 30 °C or 35 °C. Control oocytes (non-cRNA injected oocytes) treated with ChT at 35 °C showed no response (data not shown). In TRPV2-expressing oocytes, ChT treatment at 25 °C and 30 °C also did not enhance heat-evoked currents. Meanwhile, ChT treatment at 35 °C (lower than body temperature) lead to a 4-fold increase in heat-evoked currents relative to that seen for 25 °C and 30 °C (Fig. 2A, C). No heat-evoked currents were induced at 35 °C without ChT treatment (Fig. 2 B, C). These results suggest that ChT sensitizes TRPV2 to heat in the *Xenopus* oocyte expression system, and oxidation reduced the temperature threshold for TRPV2 activation to close to physiological body temperature, similar to previously reported results obtained with the mammalian cell expression system [29].

### 3.3 ChT does not enhance TRPV2 sensitivity to mechanical stimulation

**Fig. 3:**
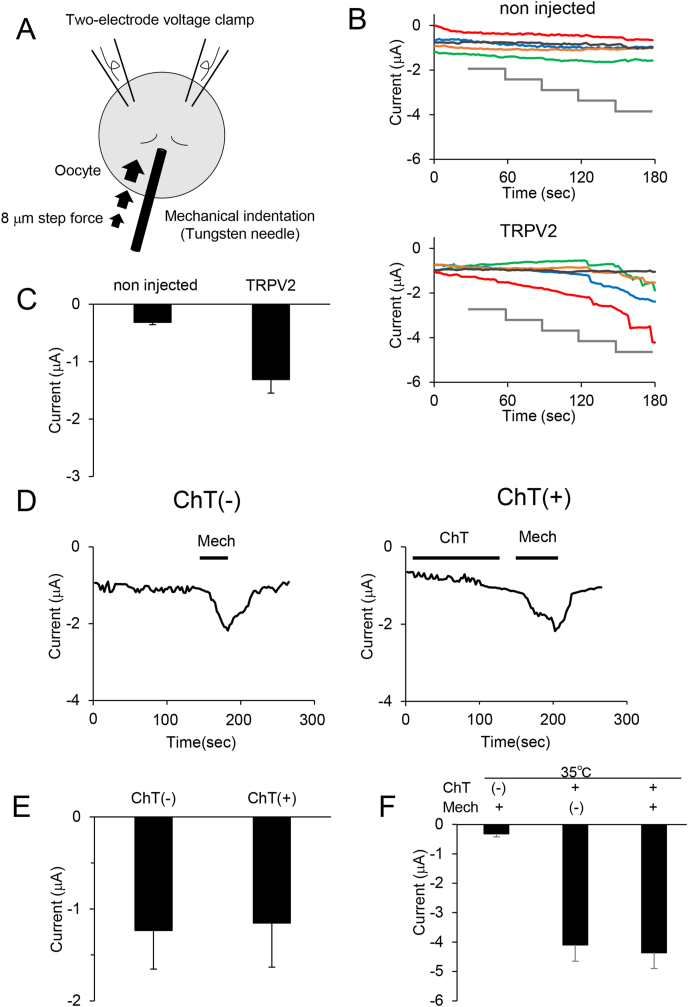
Effect of ChT on mechanical sensitivity of TRPV2-expressing oocytes. (A) Schematic depiction of application of mechanical indentation to oocytes and recording of mechanically-evoked currents with a two-electrode voltage clamp. (B) Time course of currents evoked by mechanical indentation of individual oocytes (represented by different colored traces) expressing TRPV2. The gray line represents mechanical indentation applied stepwise (8 μm each) to the oocyte every 30 s. Measurements were collected at room temperature. (C) Average current 180 s after application of mechanical indentation. Bars represent mean ± SE (n = 5). (D) Representative traces of mechanical stimuli-evoked currents in *Xenopus* oocytes expressing TRPV2 after treatment with 1 mM ChT. Mechanical stimulation (32 μm poking) was applied (shown as black line and Mech). (E) Peak average currents by application of the mechanical indentation (32 μm poking) without or with ChT treatments. Bars represent mean ± SE (n = 5). (F) Peak average currents for weak mechanical indentation (16 μm poking) at 35 °C without ChT treatment (left bar), after ChT treatment at 35 °C without (center bar) and with (right bar) weak mechanical indentation. Bars represent mean ± SE (n = 5).

We previously reported that TRPV2 is activated by mechanical stimulation [18,19]. To examine TRPV2 activation following mechanical stimulation in *Xenopus* oocytes expressing TRPV2, we designed a novel mechanical indentation system in which we generated an artificial mechanical indentation by pushing oocytes with a tungsten needle and analyzing mechanically-evoked currents (Fig. 3A). For the poking assay, mechanical stimulation was applied using a tungsten needle (outer diameter, 40 μm) mounted on the manipulator (NARISHIGE NMN-21) as described above. We rotated the dial (Z-axis) of manipulator (2.5 rotation), and the needle was then moved toward the oocyte as the 8 μm step. We observed significant mechanically-evoked current in TRPV2-expressing oocytes depending on the poking strength, but not in non-cRNA injected oocytes, indicating that TRPV2 was activated by mechanical stimulation in the *Xenopus* oocyte expression system (Fig. 3B and C).

Next, we examined the effects of ChT on the mechanical stimulation-activated TRPV2 currents. We applied middle mechanical indentation (32 μm movement). The mechanical stimulus evoked large currents (Fig. 3D). Elimination of the mechanical stimulus perfectly recovered the currents (Fig. 3D). These results indicate that our poking assay system can reversibly activate TRPV2 in *Xenopus* oocytes. Application of ChT did not change the amplitude of mechanical stimulation-activated TRPV2 current (Fig. 3D and E). These results indicate that ChT cannot sensitize mechanical sensitivity of TRPV2.

We hypothesized that TRPV2 might be synergistically activated by weak mechanical indentation after treatment with ChT below the sub-threshold concentration needed for heat stimulus. Upon application of small mechanical indentation (16 μm movement) after ChT treatment of TRPV2-expressing oocytes, we observed no mechanically-evoked currents at 25 °C (data not shown). At 30 °C, the sub-threshold for heat stimulus, ChT treatment also did not potentiate mechanically-evoked currents (data not shown). Since 30 °C may also be insufficient to potentiate TRPV2 mechano-sensitivity, we then performed these experiments at 35 °C (Fig. 3F). In the absence of ChT treatment, no TRPV2 activation was observed (Fig. 3F, left bar), but in the presence of ChT, heat-activated TRPV2 currents were evoked consistent with Fig. 2 (Fig. 3F, middle bar). Combinational application of 35 °C heat and small mechanical indentation (16 μm movement) with ChT treatment failed to potentiate TRPV2 activation (Fig. 3F, right bar). These results suggest that ChT sensitizes TRPV2 channel to heat and chemical stimuli (Fig. 1, Fig. 2) but not to mechanical stimuli (Fig. 3).

## 4 Discussion

In the present study, we demonstrated that ChT sensitizes TRPV2 sensitivity to 2-APB and heat when TRPV2 is expressed in *Xenopus* oocytes, which have a different plasma membrane composition from mammalian cells (Fig. 1, Fig. 2). Based on these findings and our previous report that TRPV2 activates in response to membrane stretch in developing neurons [18], we hypothesized that oxidation could potentiate mechanical sensitivity of TRPV2. Here we designed a new mechanical indentation method for use in a *Xenopus* oocyte expression system. Using this system, we found that the oxidant ChT did not sensitize mechanical sensitivity of TRPV2 (Fig. 3). Thus, we demonstrated for the first time that oxidation enhances sensitivity of TRPV2, a redox-sensitive TRP channel, to chemical and heat stimuli, but not to mechanical stimuli. To exert TRPV2 function, endogenous TRPV2 activation by body temperature might be occurred through the oxidized modification. Furthermore, we showed that this mechanical indentation system together with the two-electrode voltage-clamp method in a *Xenopus* oocyte expression system is a useful analytical approach to investigate the effects of oxidation on redox-sensitive TRP channels in response to various stimuli.

We showed that mechanical indentation evoked TRPV2 currents in the *Xenopus* oocyte expression system (Fig. 3B–D), but these currents varied among oocytes (Fig. 3B). The mechanical indentation evoked TRPV2 currents seemed to be very slow, since it takes for 30 s that the needle (to apply stimuli) moved 8 μm. It might be possible that the TRPV2 currents slowly increased depending on the increase of poking strength. We reported that TRPV2 accumulated to the regions where mechanical stimuli generated [19]. Thus, it might be also possible that the TRPV2 clustering, in the regions where mechanical stimuli generated, also slowly increased depending on the increase of poking strength. Moreover, in the *Xenopus* oocyte expression system ChT did not show temperature-dependent enhancement of TRPV2 sensitivity, as evidenced by the lack of effect of mechanical stimulation at 35 °C (Fig. 3F). This outcome raises two possibilities: (i) the magnitude of the mechanical stimulus on the membrane was too small and more intense stimulation may be needed for oxidation-dependent enhancement of mechanical sensitivity; and/or (ii) differences in membranes of *Xenopus* and mammalian cell plasma membranes could affect channel activity. The main method to analyze response of ion channels to mechanical stimulation involves whole-cell patch clamping wherein mechanical indentation is applied using a blunt glass probe controlled by a piezo electric device or a cell-attached patch clamp to measure the current when negative pressure is applied using a high-speed pressure clamp through a patch pipette in cells expressing the protein of interest, such as HEK293T cells [32,33]. The two-electrode voltage clamp recording in *Xenopus* oocyte expression system is better-suited for high throughput analysis [34,35], and thus our system could have future applications to screen drugs that target TRPV2, or for biophysical studies on this channel.

**Fig. 4:**
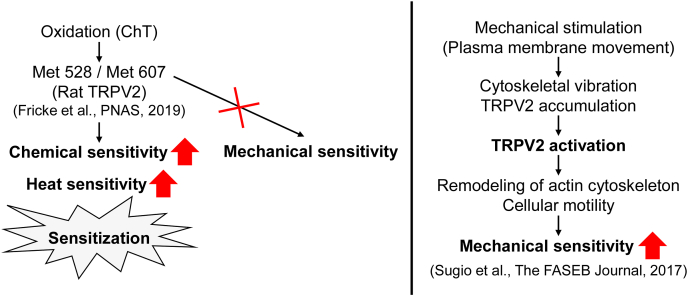
Mechanism of TRPV2 activation in response to chemical ligand, heat and mechanical stimulation in the presence of oxidation. ChT oxidizes TRPV2 methionine residues (M528 and M607 in rat TRPV2). This oxidation is predicted to potentiate TRPV2 sensitivity to chemical and heat stimulation. ChT does not potentiate TRPV2 sensitivity to mechanical stimulation, which could instead affect cytoskeletal architecture (e.g., organization of actin filaments) to induce TRPV2 localization to the plasma membrane. This plasma membrane localization can promote TRPV2 activation that triggers further remodeling of the actin cytoskeleton and enhancement of cellular motility. Finally, TRPV2 activation potentiates mechanical sensitivity according to our previous finding.

Concerning the mechanism by which TRPV2 is activated by chemical, heat and mechanical stimulation in response to oxidation in *Xenopus* oocytes, Fricke et al. reported that oxidation of Met residues (M528 and M607 in rat TRPV2) contributes to enhanced sensitivity to chemical ligand and heat [29]. Similar to mammalian cells, *Xenopus* oocytes expressing TRPV2 may also have enhanced sensitivity to chemical ligands and heat due to Met residue oxidation (Fig. 4, left side). However, the inability of ChT to enhance TRPV2 mechanical sensitivity (Fig. 3), suggests that the activation mechanism for potentiating mechanical sensitivity may differ from that involving methionine oxidation. In our previous analysis using PC12 cells, we reported that plasma membrane movement perturbs the cytoskeletal architecture (e.g., changes in actin filament organization) and subsequent perturbations induce TRPV2 accumulation and activation at the plasma membrane. TRPV2 activation triggers remodeling of the actin cytoskeleton and enhances cellular motility. Finally, TRPV2 activation can promote TRPV2 sensitivity to mechanical stimulation (Fig. 4, right side). Thus, this pathway likely does not involve oxidation of Met residues (M528 and M607) promoted by ChT treatment and, as such, suggests that oxidation is not related to the potentiation of TRPV2 activation in response to the mechanical stimulus.

## Funding

This study was supported by Grants-in-Aid for Scientific Research from the 10.13039/100007449Takeda Science Foundation, 10.13039/100008608Sumitomo Foundation, The 10.13039/100009666Salt Science Research Foundation (No.2129), 10.13039/100017103Urakami Foundation for Food and Food Culture Promotion, Narishige Neuroscience Research Foundation, Takano Life Science Research Foundation and 10.13039/501100001700MEXT/10.13039/501100001691JSPS KAKENHI JP21H05632 (Glia Decoding), JP18H03124, JP18K19418 (all to K.S.).

## Declaration of competing interest

The authors declare no competing interests.
